# Lessons from the field: compound-specific management in acute pesticide poisoning

**DOI:** 10.1093/trstmh/trae003

**Published:** 2024-02-10

**Authors:** Vasundhara R Verma, Thomas Lamb, Md Abdus Sattar, Aniruddha Ghose, Michael Eddleston

**Affiliations:** Pharmacology, Toxicology and Therapeutics, University/BHF Centre for Cardiovascular Science, University of Edinburgh, UK; Pharmacology, Toxicology and Therapeutics, University/BHF Centre for Cardiovascular Science, University of Edinburgh, UK; Department of Medicine, Chittagong Medical College and Hospital, Chattogram, Bangladesh; Department of Medicine, Chittagong Medical College and Hospital, Chattogram, Bangladesh; Pharmacology, Toxicology and Therapeutics, University/BHF Centre for Cardiovascular Science, University of Edinburgh, UK; South Asian Clinical Toxicology Research Collaboration, University of Peradeniya, Peradeniya, Sri Lanka; Centre for Pesticide Suicide Prevention, University of Edinburgh, UK

**Keywords:** insecticide, organophosphate, pesticide, poisoning, pyrethroid, toxicology

## Abstract

Pesticide poisoning is a common medical emergency in the rural tropics, with significant associated mortality. Pesticide poisoning is an umbrella term that encompasses a wide variety of substances with differing clinical toxidromes and outcomes. Despite this, confirmation of the specific compound ingested is rarely performed. In this Lessons from the Field, we argue that pesticide-specific management is integral to optimise management. Using data from a quality improvement project in Chittagong, Bangladesh, we demonstrate that identifying the specific compound is possible in most patients through careful history taking and examination of the pesticide bottle. Identification of the specific compound is essential for anticipating and reducing complications, administering appropriate and timely management and reducing the length of hospital stay and cost of unnecessary medical intervention.

Pesticide poisoning is a major global health problem. Each year there are an estimated 110 000–168 000 deaths resulting from acute self-poisoning with pesticides.^1^ Most deaths occur due to deliberate self-poisoning in agricultural communities of Africa and Asia. Following the successful introduction of pesticide legislation and the banning of class 1 highly hazardous pesticides in some countries, there has been a reduction in pesticide poisoning–related mortality and increasing diversification of pesticide use.^2^ This increased diversification of pesticide use has resulted in a greater variety of pesticides responsible for poisonings.

Pesticides used for deliberate self-poisoning result in a wide spectrum of clinical toxidromes. Clinical outcomes can range from having minimal symptoms with an excellent prognosis to severe cholinergic crisis including bronchospasm, bronchorrhoea, miosis, sweating, agitation and muscle paralysis causing high case fatality rates. Within pesticide classes such as organophosphate (OP) insecticides, there is considerable heterogeneity in clinical features, complications and outcomes dependent on which specific OP compound is ingested.^3^ For example, dimethoate OP poisoning typically causes an acute cholinergic crisis and cardiovascular instability. This differs markedly from poisoning from highly lipid soluble OP compounds such as fenthion, which causes fewer cholinergic features and less cardiovascular instability but occasional delayed neuromuscular paralysis.^3^ This heterogeneity is further complicated by variations in formulation (powder, granules and liquid), pesticide concentration, solvents, emulsification and mixed compounds that can influence ingested dose and absorption.

Despite variations in clinical manifestations, complications and outcomes, many patients with a history of pesticide ingestion present with similar clinical features, including vomiting, miosis and reduced conscious level.^4^ This can make a compound-specific diagnosis based on toxidrome alone challenging. Due to historically high rates of OP poisoning and associated high case fatality rates, patients are commonly treated empirically with OP-specific treatments including atropine and pralidoxime.^5^

During a 3-month period from November 2015 to February 2016, patients presenting to Chittagong Medical College, Bangladesh with a history of pesticide poisoning were interviewed within 24 h of admission as part of a quality improvement project. The compound ingested was identified from the history provided by the patient or relatives, examination of the pesticide bottle or a photograph of the pesticide bottle. The confirmed compound was then compared with the clinical diagnosis made on admission by the attending medical team in which specific compounds were rarely identified. The clinical teams were notified of circumstances in which the clinical diagnosis differed from that determined by the research team.

During the observation period, 314 patients presented with a history of pesticide ingestion. The specific compound was identified from the history or bottle in 221/314 (70.4%) patients (Table 1). Of the 314 patients, the majority (283 [90.1%]) were initially diagnosed and managed as OP or carbamate poisoning based on clinical presentation. In patients empirically managed as OP/carbamate poisoning, the compound ingested was subsequently identified by history or the bottle in 198 patients. A total of 63/198 patients (31.8%) had ingested other insecticides and were therefore misdiagnosed based on clinical toxidrome. All 63 patients received unnecessary gastric lavage, intravenous pralidoxime and atropine (median dose 9 mg [interquartile range 6–12]).

**Table 1.** Pesticides ingested among those with confirmation of compound ascertained by history or bottle.

Substance ingested	Patients, n (%)^a^	Specific pesticides
OP insecticides^b^	110 (49.8)	Chlorpyrifos 30, dimethoate 28, malathion 26, phenthoate 11, diazinon 8, quinalphos 6
Carbamate	10 (4.5)	Carbofuran 6, carbosulfan 4
OP and pyrethroid combination insecticide	21 (9.5)	Chlorpyrifos+cypermethrin 20, profenofos+cypermethrin 2
Pyrethroid	52 (23.5)	Cypermethrin 23, lambda-cyhalothrin 21, fenvalerate 4, permethrin 4
Other insecticide	13 (5.9)	Abamectin 6, emamectin benzoate 4, ivermectin 2, avermectin 1
Fungicide	9 (4.1)	Hexaconazole 4, azoxystrobin + difenoconazole 2, copper oxychloride 1, mancozeb 1, propiconazole + tricyclazole 1
Herbicide	4 (1.8)	Paraquat 3, glyphosate 1
Rodenticide	2 (0.9)	Zinc phosphide 2

^a^Total patients with substance identified was 221.

^b^OP insecticide formulations: chlorpyrifos 20–48 emulsifying concentrate (EC), dimethoate 40 EC, malathion 57 EC, phenthoate 50 EC, diazinon 10 granules, quinalphos 25 EC.

Pesticide poisoning is common and often neglected. Clinical manifestations vary considerably, both between and within pesticide groups, necessitating individualised care. In this quality improvement project and through our personal experience of managing acute pesticide poisoning, identification of the specific compound and formulation is possible in most patients but rarely performed. Identification is possible through history and examination of the pesticide bottle without the need for complex diagnostic equipment or toxicological analysis. Identification is made easier when using a national database of locally available compounds with brand names. This method of pesticide identification has been found to correlate with toxicological analysis in >80% of cases.^3,6^ Identifying the specific compound and formulation ingested allows the attending clinician to deliver appropriate emergency care, anticipate potential complications, improve prognostication and ultimately deliver patient-centred care.

As demonstrated in this study, there is a tendency to overdiagnose OP poisoning and initiate emergency treatment, including decontamination of gastric contents using nasogastric lavage and OP-specific antidotes. Acute pyrethroid poisoning can often mimic OP poisoning, with similarities in the toxidrome including vomiting, miosis and reduced consciousness.^4^ A misdiagnosis of pyrethroid or other pesticide poisoning as OP poisoning exposes patients to potentially harmful medical interventions. Furthermore, these interventions place huge additional financial burdens on patients and increase the hospital length of stay.^5^

In addition to helping guide emergency management, identification of specific compounds may help clinicians anticipate complications and guide prognosis. Highly lipid-soluble OP compounds such as fenthion have been associated with delayed-onset respiratory failure and patients may benefit from enhanced observation and frequent assessment of tidal volume to detect imminent respiratory failure. Paraquat, a non-selective herbicide, can cause progressive irreversible pulmonary renal syndrome. Appreciation of its progressive nature and poor outcome may help clinicians understand the limitations of ventilation in patients with respiratory failure.^6^

## Conclusions

Emergency management of patients presenting with acute pesticide poisoning should include both clinical assessment of the toxidrome and an attempt to identify the specific compound from history, the bottle or photographs. Identification of the specific compound ingested from self-poisoning is possible in most patients and is key to predicting and reducing complications, administering appropriate and timely management and reducing the length of hospital stay and cost from unnecessary medical interventions.

## Data Availability

Anonymised data available on request.
